# Studying DNA Double-Strand Break Repair: An Ever-Growing Toolbox

**DOI:** 10.3389/fmolb.2020.00024

**Published:** 2020-02-21

**Authors:** Alexandra C. Vítor, Pablo Huertas, Gaëlle Legube, Sérgio F. de Almeida

**Affiliations:** ^1^Instituto de Medicina Molecular João Lobo Antunes, Faculdade de Medicina da Universidade de Lisboa, Lisbon, Portugal; ^2^Department of Genetics, University of Seville, Seville, Spain; ^3^Centro Andaluz de Biología Molecular y Medicina Regenerativa-CABIMER, Universidad de Sevilla-CSIC-Universidad Pablo de Olavide, Seville, Spain; ^4^LBCMCP, Centre de Biologie Integrative (CBI), CNRS, Université de Toulouse, Toulouse, France

**Keywords:** DNA repair, homologous recombination (HR), non-homologous DNA end joining, chromatin, DNA damage

## Abstract

To ward off against the catastrophic consequences of persistent DNA double-strand breaks (DSBs), eukaryotic cells have developed a set of complex signaling networks that detect these DNA lesions, orchestrate cell cycle checkpoints and ultimately lead to their repair. Collectively, these signaling networks comprise the DNA damage response (DDR). The current knowledge of the molecular determinants and mechanistic details of the DDR owes greatly to the continuous development of ground-breaking experimental tools that couple the controlled induction of DSBs at distinct genomic positions with assays and reporters to investigate DNA repair pathways, their impact on other DNA-templated processes and the specific contribution of the chromatin environment. In this review, we present these tools, discuss their pros and cons and illustrate their contribution to our current understanding of the DDR.

## Dna Double-Strand Break Detection, Signaling and Repair

DNA double-strand breaks (DSBs) are the most cytotoxic DNA lesions. Their detection, signaling, and repair require a comprehensive cellular response collectively known as the DNA damage response (DDR). The DDR requires the activation of the ATM kinase, a member of the phosphoinositide 3-kinase (PI3K)-related protein kinase family (Blackford and Jackson, 2017), which is rapidly recruited to chromatin in response to DSBs through the interaction with the MRE11-RAD50-NBS1 (MRN) complex (van den Bosch et al., 2003). This recruitment triggers the phosphorylation of a large number of substrates to initiate a signaling cascade that activates cell cycle checkpoints and promotes the recruitment of repair factors to the damage site. One of the substrates of ATM kinase activity is the serine 139 of the carboxyl terminus of the histone variant H2AX, which in its phosphorylated version is referred to as γH2AX (Burma et al., 2001). Once established, γH2AX promotes the recruitment of additional ATM molecules and the sequential accumulation of other DDR proteins, creating a positive feedback loop that fuels further spreading of γH2AX (van Attikum and Gasser, 2009; Polo and Jackson, 2011; Shi and Oberdoerffer, 2012).

**FIGURE 1:**
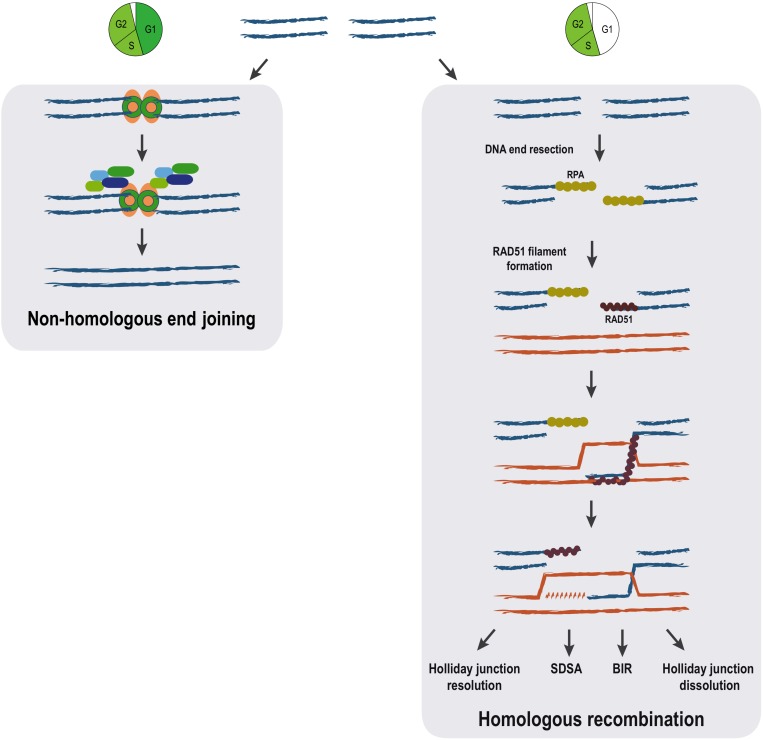
Pathways for DSB repair. The main steps of non-homologous end joining and homologous recombination repair mechanisms are represented. Homologous recombination can proceed through distinct pathways (holiday junction resolution, synthesis-dependent strand annealing (SDSA), break-induced replication (BIR) and holiday junction dissolution) all sharing identical initial steps. The cell cycle is a major determinant of the choice between the DSB repair mechanisms. While NHEJ is available throughout interphase, homologous recombination pathways are restricted to S/G2 phases of the cell cycle.

DNA double-strand breaks repair can be achieved by different means that are commonly grouped in two broad categories depending on the use or not of a homologous DNA sequence as a template. Repair by non-homologous end joining (NHEJ) involves direct resealing of the two broken ends independently of sequence homology. Although being active throughout the cell cycle, NHEJ is relatively more important during G1 (Chang et al., 2017). A scheme showing the most important steps of NHEJ is shown in Figure 1; Chang et al., 2017). NHEJ represents the simplest and fastest mechanism to heal a DSB, thus it is the most predominant DSB repair pathway within the majority of mammalian cells, even though it may occasionally lead to loss of genetic information (Chang et al., 2017).

In contrast to NHEJ, homologous recombination (HR) requires a homologous DNA sequence to serve as a template for DNA-synthesis-dependent repair and involves extensive DNA-end processing (Huertas, 2010). As expected, HR is extremely accurate, as it leads to precise repair of the damaged locus using DNA sequences homologous to the broken ends. HR predominantly uses the sister chromatid as a template for DSB repair, rather than the homologous chromosome (Johnson, 2000). This tight regulation is ensured thanks to both a strong inhibition of HR during G1 when a sister chromatid is absent (Hustedt and Durocher, 2016), but also thanks to the nature of the newly replicated chromatin, which favors HR (Saredi et al., 2016; Pellegrino et al., 2017; Nakamura et al., 2019). The key first step in HR, determinant for DSB pathway choice, is 5′ to 3′ resection: the processing of the 5′ DNA strand at the DSB by multiple nucleases and accessory proteins, resulting in 3′ single-stranded DNA (ssDNA) (Huertas, 2010; Symington, 2014). The 3′ ssDNA stretches created during resection are used for template search and recombination (Figure 1).

As described above, both HR and NHEJ safeguard genome integrity and proceed through a cascade of events whereby DNA damage sensors, transducers, and effectors detect and rejoin the broken DNA ends (Harper and Elledge, 2007). All these events take place within the chromatin environment, which is the actual substrate for the repair machinery. While the past 50 years have seen a mounting understanding of the DDR pathways, the contribution of the chromatin environment and nuclear organization to genome stability, particularly how it is organized upon the interplay between the DDR and the other cellular processes, has only begun to emerge over the past decade. Chromatin is modified *in cis* to the DSB and this break-induced chromatin landscape contributes to recruiting DNA repair factors, thanks to interactions between histone modifications and their readers (e.g., 53BP1 interacts with nucleosomes bearing H2AK15ub and H4K20me2). In addition, during DSB repair, the destabilization of nucleosomes further enhances accessibility and regulate the mobility of the broken DNA ends (Clouaire and Legube, 2019). Moreover, the original chromatin landscape of the damaged locus also contributes to the decision between DSB repair pathways (Clouaire and Legube, 2015; Fortuny and Polo, 2018; Bartke and Groth, 2019).

Most of our ever-growing knowledge of the DDR and, in particular, the DSB repair mechanisms has been possible due to a set of techniques that have allowed us to create DSBs in a programed manner. In this review we are coming back on those methodologies that have recently fostered our capacity to accurately study the full complexity of repair mechanisms, allowing us to consider the genomic position of the DSB and the contribution of chromatin, as well as their crosstalk with other DNA-templated processes.

## Inducing Dsbs at Random Locations

Historically, the study of the DDR relied mostly on the artificial induction of DSBs by either chemical or physical agents stochastically throughout the genome. The genomic location of these DSBs is not homogenous in the cell population and is poorly controlled. Importantly, the number of breaks can be modulated by adjusting either the dose or the duration of the treatments. Moreover, the stochastic induction of DSBs is usually very fast, requiring seconds or a few minutes, facilitating downstream kinetic studies.

### Ionizing Radiation-Induced Breaks

**FIGURE 2:**
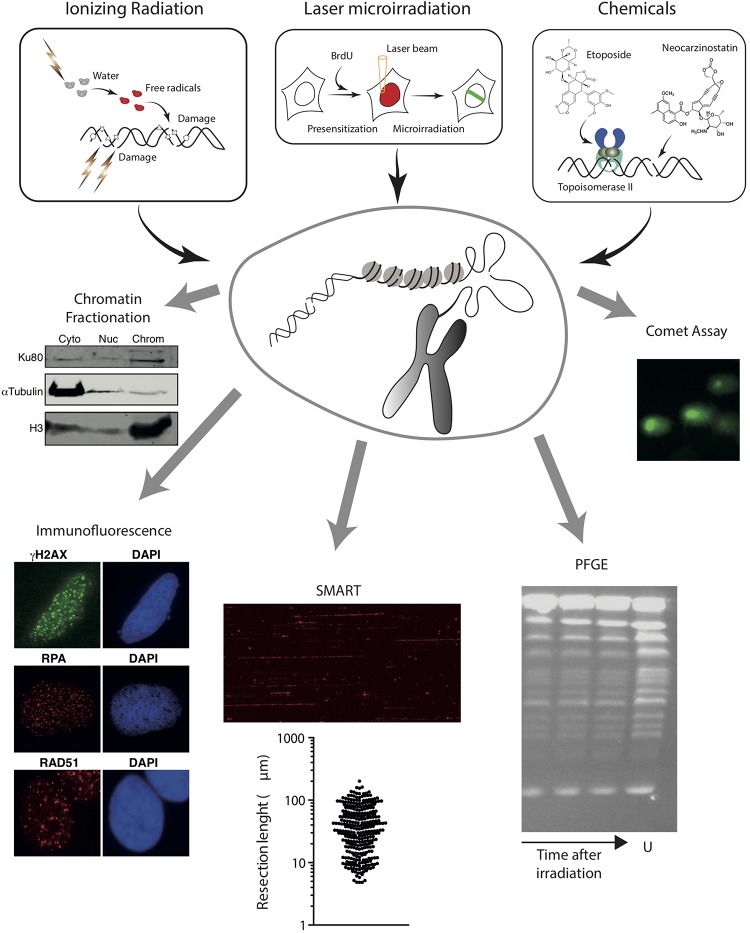
Schematic overview of methods to induce random DNA breaks in the genome using radiation (top left) or chemical agents (top right). The energy of radiation can be transferred directly to the DNA molecule or can ionize other molecules like water that will then attack the DNA. In addition to DNA breaks, radiation damage induces additional modifications on the DNA, represented as stars, pentagons or triangles. Examples of chemical induction of DSBs by the direct attack of DNA (using drugs such as neocarzinostatin) or indirectly by affecting specific proteins (Etoposide inhibits the topoisomerase cycle) are shown. Experimental approaches that can be coupled with these methods to induce DSBs are also represented in the bottom. From left to right, chromatin fractionation, to observe the accumulation of a protein on the cytoplasmic (Cyto), nucleoplasmic (Nuc), or chromatin (Chrom) fractions; Immunofluorescence, to visualize the formation of nuclear foci using specific antibodies; SMART, to measure the length of resected DNA; PFGE, to visualize the presence of pieces of broken chromatin (in the figure, S. cerevisiae chromosomes untreatated (U) or at different times upon irradiation); and comet assay, to study the appearance of breaks at the single-cell level. For details, see the main text.

The exposure of cells to a source of ionizing radiation (IR) causes the appearance of a plethora of different genomic lesions (Kavanagh et al., 2013). They can arise from the radiation directly hitting the DNA, or indirectly by the effect of radiation-induced reactive species resulting from the ionization of several molecules, including water (Figure 2). The source of the DNA lesions depends on the type of radiation. For example, X-rays induce DNA damage mainly through indirect effects, whereas heavy particles, such as protons, interact more directly with the DNA backbone. Importantly, radiation creates many types of damage on the DNA, including all kinds of base modifications, loss of bases, single-strand breaks (SSBs) or DSBs. Indeed, it has been estimated that IR produces ten times more SSBs than DSBs (Ma et al., 2012). The degree of heterogeneity of the lesions created by IR also depends on the nature of the radiation, mostly on its LET (linear energy transfer: the amount of energy that the particle transfers to the medium along its trajectory per distance unit) (Zirkle and Tobias, 1953). In any case, all different types of DNA damage are quickly repaired, except for DNA breaks. DSBs formed upon ionizing radiation exposure are normally clustered SSBs, i.e., usually formed when two DNA lesions appear in opposite strands in close proximity (<10 bp) (Milligan et al., 1995). The broken DNA ends produced by radiation usually show chemical alterations, being considered “dirty” ends (Weinfeld and Soderlind, 1991). While IR induces breaks stochastically all over the genome, the randomness also depends on the LET of the radiation. Indeed, high LET particles tend to produce clusters of DSBs in close proximity (Löbrich et al., 1996; Newman et al., 1997). Additionally, high LET radiation seems to induce DSBs less randomly than photons in high-order chromatin structures (Radulescu et al., 2006).

Of interest, upon DSB induction following exposure to radiation, many DDR factors tend to accumulate temporarily at sites of DNA damage, forming the so-called Ionizing Radiation-Induced Foci (IRIF) (Ciccia and Elledge, 2010; Polo and Jackson, 2011). Importantly, some of these, including γH2AX, can spread over megabases along the DNA flanking the break (Iacovoni et al., 2010). If combined with the use of specific antibodies or fluorescent tagged-versions, this strong regional concentration allows for the visualization of IRIF under a fluorescent microscope (Figure 2). Hence IRIF formation has been and still is, one of the easiest and most used tools to study the recruitment of DNA repair factors during the DDR. Additionally, since some of them, such as γH2AX foci, appear specifically in response to a DNA lesion and disappear when the repair process has been completed, clearance of IRIF provides a simple way to analyze the kinetics of DNA repair (Bouquet et al., 2006).

Analysis of IRIF can also inform on the DNA repair pathway choice. For instance, early steps of HR can be observed by the accumulation of the MRN complex or CtIP that will in turn be responsible of the formation of ssDNA (Mirzoeva and Petrini, 2001; Sartori et al., 2007). Resection products can also be observed by the accumulation of RPA (Sartori et al., 2007; Cruz-García et al., 2014; López-Saavedra et al., 2016; Figure 2). An alternative is the observation of BrdU-labeled ssDNA using non-denaturing conditions in cells treated with this thymidine analog for one cell cycle to ensure that one DNA strand is completely labeled in all chromosomes (Sartori et al., 2007). For later DNA repair steps, RAD51 accumulation is the preferred marker of recombination (Mirzoeva and Petrini, 2001; Figure 2). NHEJ proteins, however, are difficult to see at DNA damage foci due to the low number of units bound to each DSB and the high background levels. Thus, specific protocols have been developed for their observation (Britton et al., 2013). Alternatively, other accessory factors of NHEJ and HR, such as 53BP1 or BRCA1, respectively, can be used as a proxy for these DNA repair pathways (Chapman et al., 2013; Escribano-Díaz et al., 2013; Zimmermann et al., 2013). Additionally, for low abundant factors, the signal can be boosted by using a Proximity Ligation Assay (PLA) to visualize if our protein or specific post-translational modification of interest is in close proximity to factors/modifications known to enrich at DSBs, such as γH2AX (Gullberg et al., 2003). A recent variation of the PLA, the DNA damage *in situ* ligation followed by proximity ligation assay (DI-PLA), allows detection and imaging of individual DSBs in cells (Galbiati et al., 2017).

If immunofluorescence analysis of IRIF is not appropriate (for instance, due to lack of antibodies or low amount of protein at DNA breaks precluding the observation of a positive signal under the microscope), the binding, recruitment, retention or release of specific proteins can be studied using a chromatin fractionation approach (Figure 2). First used to analyze the recruitment of NHEJ factors (Drouet et al., 2005), it can be adapted for any factor if there are specific antibodies that work in western blot. Briefly, chromatin fractionation consists in the separation of cytosolic, nucleoplasmic, and chromatin fractions from undamaged and radiation-exposed cells. The resolution of the proteins in SDS-PAGE followed by western blotting using appropriate antibodies from samples collected at different time-points upon DNA damage uncovers the dynamics of recruitment/retention/release of the studied factors. This method can be combined with depletion or inhibition of specific proteins, therefore uncovering the hierarchy of recruitment of different DNA repair factors to DSBs.

DNA resection can be specifically investigated with high resolution using single-molecule analysis of resection tracks (SMARTs). This is a modified DNA combing approach, in which resection of broken DNA ends leads to the exposure of otherwise inaccessible BrdU-epitopes previously incorporated in the DNA. When combined with an immunodetection protocol using fluorescence microscopy, SMARTs allows the direct visualization and quantification of individual tracks of resected DNA after IR (Cruz-García et al., 2014; Huertas and Cruz-Garcia, 2018; Figure 2).

Finally, approaches such as pulsed-field gel electrophoresis (PGFE) or single-cell gel electrophoresis (also known as comet assay) can also be combined with IR exposure to directly investigate DSB repair (Figure 2). IR-induced DSBs fragment the genome in smaller portions, which can be measured using PFGE to estimate the number and repair of DNA breaks. This technique allows the separation of rather large DNA pieces by forcing them to pass through an agarose matrix in response to changing electric fields (Schwartz and Cantor, 1984; Carle and Olson, 1985). Yeast chromosomes are small enough to be resolved in PFGE (Schwartz and Cantor, 1984; Carle and Olson, 1985; Figure 2), thus fragmentation due to DNA damage can be observed by the appearance of a smear of smaller bands (Contopoulou et al., 1987). The much larger mammalian chromosomes, on the contrary, remain on the wells during PFGE, and only smaller fragments caused by random DSBs will enter the gel (Ager et al., 1990). The size distribution of the DNA portions is dependent on the number of breaks. Thus, PFGE reveals the appearance of DSBs and estimates their number. Moreover, by taking samples at fixed times after exposure to a DNA damaging source, PFGE can be used to quantify the kinetics of DNA repair. A variation of this technique was developed in the Resnick laboratory using circular chromosomes in yeast or Epstein-Barr virus episomes in human cells (Ma et al., 2008, 2012). Another variation of this technique, the single-cell gel electrophoresis or comet assay, is a convenient way to estimate the number of DSBs created upon a given treatment with DNA damaging agents, such as IR, and to follow the kinetics of DNA repair in individual cells. Comet assays can be performed using either neutral or alkaline buffers to focus on DSBs or SSBs, respectively. Briefly, cells are treated with the DNA damage source, embedded in agarose to retain the nuclear structure, lysed and subjected to electrophoresis (Olive et al., 1991). DNA is attracted to the anode, but only broken fragments are small enough to abandon the nucleus (Figure 2). After staining with a DNA dye, nuclei are observed with a fluorescent microscope and the displacement of DNA from the nucleus depends on the number of breaks per genome (Olive et al., 1991). By analyzing samples at different time points after DSB induction, the kinetics of repair can be estimated.

#### Key Points

**(+)** Radiation exposure provides an easy and robust way to analyze the recruitment of proteins to sites of DNA damage (provided that their level of binding is high enough) and to study the DNA repair kinetics using different approaches (e.g., γH2AX foci disappearance, comet assays, or PGFE).

**(−)** Radiation not only induces DSBs but also a plethora of other damages in the cell, and creates “dirty” ends, mostly in a random manner on the genome, hence likely biased toward the untranscribed genome in higher eukaryotes (given that genes represent a minority of the mammalian genome). Moreover, since radiation induces DSBs at unknown locations, and in a non-homogenous manner in the cell population, locus-specific analyses of DDR factor recruitment or chromatin modifications using chromatin immunoprecipitation (ChIP) studies, for instance, is not possible.

**^∗^** Additionally, each readout of these stochastic DSBs has its own pros and cons. For example, ssDNA observed by SMART, RPA or BrdU foci might reflect unwound DNA; the COMET assay also detects apoptotic cells, albeit the tail shape is different; chromatin accumulation of some factors might occur independently of DNA damage and in response to other signals. Thus, in all cases, appropriate controls must be used.

### Non-ionizing Radiation: Laser Beams

In addition to IR-induced DNA damage, in which cells are exposed to an X-ray lamp or a Cesium irradiator, non-ionizing radiation can also be used to study DSBs. For instance, ultraviolet A (UVA) radiation can be used to create hundreds of DSBs along the path of a laser beam (line or spot) through laser scanning microscopy (Lukas et al., 2003). UVA does not directly generate DSBs. However, pre-treatment of cells with the thymidine analog BrdU for one cell cycle to allow its incorporation in one DNA strand, sensitizes DNA to UVA, causing the appearance of clustered SSBs and DSBs along the laser beam track. Laser irradiation provides two main advantages. First, one can decide where to direct the laser beam in the nucleus, allowing to target specific subnuclear compartments, such as the nucleolus (Kruhlak et al., 2007). Second, the concentration of hundreds of breaks along a laser track facilitates the observation of the recruitment of factors that either do not spread at all, or gradually increase over time, and for which foci are therefore difficult to see, especially at early time points. As such, laser irradiation represents the most powerful tool to accurately determine the kinetics of DDR factors, providing a temporal resolution below 10 s.

Combined with the expression of fluorescently labeled proteins, laser microirradiation has provided unprecedented temporal resolution of the sequence of events following DNA damage (Kochan et al., 2017; Aleksandrov et al., 2018). This approach can also be complemented with FRAP and FLIP studies (see Mortusewicz and Leonhardt, 2007). The use of fluorescently-tagged histone proteins allowed the study of chromatin dynamics following damage with great resolution (Burgess et al., 2014; Luijsterburg et al., 2016; Sellou et al., 2016; Smith et al., 2018). Finally, this method has been useful to investigate the release of factors from DSBs and the post-translational modifications (PTMs) that drive such dynamics. In this case, the signal void created by the absence of the protein or by the removal of a specific PTM can be seen as a negative stripe (or anti-stripe) (Chou et al., 2010; López-Saavedra et al., 2016).

#### Key Points

**(+)** Laser microirradiation represents the best technique today to temporally resolve the sequence of events at DSBs, allowing to observe very early (<10 s) and/or transient repair proteins recruitment and chromatin modifications.

**(−)** Microirradiation induces a large number of localized, clustered DNA lesions (not only DSBs), that may also initiate specific responses. Moreover, it is neither amenable for molecular characterization of the repair outcome at the sequence level, nor for ChIP, which limits the spatial resolution that can be achieved.

### Chemically Induced Breaks

In contrast to radiation treatments, that require specialized and expensive equipment, chemical-induction of DSBs is cheap and easy to implement in any laboratory and can be coupled with almost any experimental protocol. Usually, cells are treated with a defined concentration of a chemical agent for a fixed amount of time. It is important to distinguish between acute (from minutes to a few hours) versus chronic (for days) treatments, as the responses will vary enormously. Many types of chemical agents can indirectly cause DNA breaks. For example, chemical inhibition of topoisomerases I and II causes SSB and DSB respectively (Huang et al., 2003; Figure 2). SSBs caused by camptothecin, a common inhibitor of topoisomerase I, can, in turn, be converted to DSBs during replication. Replication inhibitors, such as HU or aphidicolin, and crosslinker agents, like cisplatin or mitomycin C, can also cause one ended DSBs due to fork collapse (Saintigny et al., 2001; Noll et al., 2006). Additionally, several chemical agents imitate the effect of ionizing radiation and break the DNA directly (Figure 2). These radiomimetic drugs include bleomycin, phleomycin or neocarzinostatin (Sleigh, 1976; Edo and Koide, 1997; Chen and Stubbe, 2005).

Of importance, given their different modes of action, all the above-mentioned drugs will produce different types of DSBs: either located at different genomic regions and/or introduced during different cell-cycle stages. For instance, DSBs created by radiomimetic drugs show a bias toward specific sequences (Murray and Martin, 1985; Burden et al., 1996). Moreover, topoisomerase II poisons such as etoposide preferentially induce lesions at CTCF binding loci located close or within transcriptionally active units (Canela et al., 2017, 2019; Gothe et al., 2019). Topoisomerase I and replication inhibitors induce DSBs specifically during S phase or the following mitosis (Saintigny et al., 2001; Huang et al., 2003).

The analysis of DSBs induced by chemical agents can be performed by the same approaches described for irradiation-induced breaks (Figure 2). In addition, a number of genome-wide methodologies have been recently developed to directly map DSB distribution at a nucleotide resolution across the genomes in a cell population (Bouwman and Crosetto, 2018) including for instance Break-seq, BLESS, iBLESS, BLISS, DSB-capture, End-seq and BrlTL (Hoffman et al., 2015; Canela et al., 2016; Lensing et al., 2016; Biernacka et al., 2018; Mirzazadeh et al., 2018; Shastri et al., 2018). These techniques are well suited to investigate DSBs that occur non-randomly across the genome such as those induced by topoisomerase II poisons for instance. Of importance they not only provide an information about DSBs positions on the genome, but they are also quantitative, hence providing an estimate of break frequency in the cell population (Aymard et al., 2017; Canela et al., 2019).

#### Key Points

**(+)** Treatment with genotoxic compounds represents an easy to implement and robust way to analyze the recruitment of DSB repair factors and to study the repair kinetics using different approaches (kinetics of γH2AX foci, comet assays, and PGFE).

**(−)** Drugs produce different types of DNA damage, at different genomic loci, and most show a preference for specific cell cycle stages, which should be carefully considered during data interpretation.

## Methods to Induce Annotated DNA Breaks at Transgenic Loci Inserted in the Genome

Different labs have sought to develop tools for the site-specific induction of DNA breaks making use of restriction enzymes targeting integrated exogenous cleavage sites, otherwise absent from the genome. Such tools overcome the ambiguity of DNA lesions introduced by previous methods and allow the inspection of protein recruitment during the DDR to a site-specific DSB and the assessment of chromatin remodeling events with nucleosome resolution. Moreover, they can be combined with strategies to control the timing of DSB induction, for instance by controlling the nuclear translocation of the restriction enzyme, affording a valuable strategy to measure kinetic parameters of the DDR in live cells (Berkovich et al., 2007; Soutoglou et al., 2007).

**FIGURE 3:**
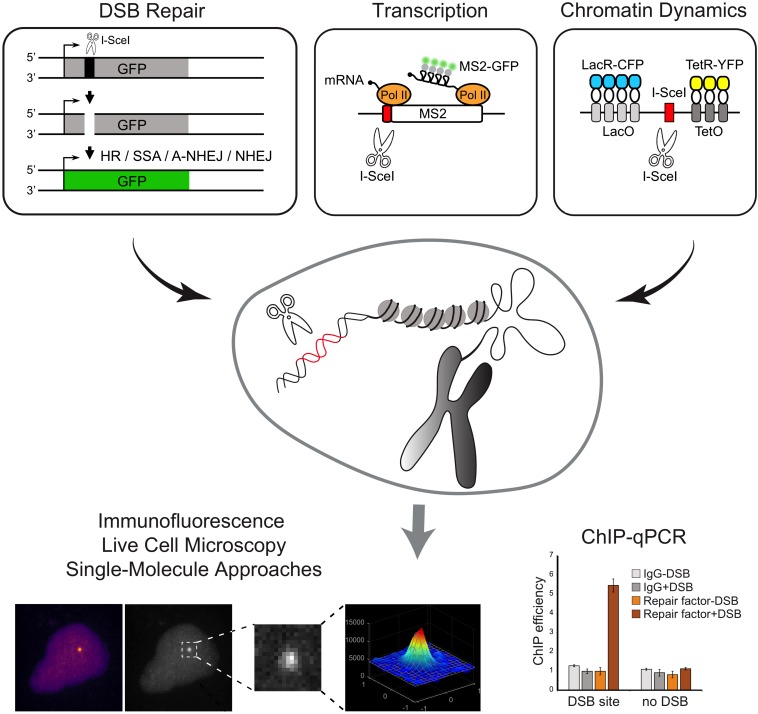
Schematic overview of methods to induce annotated DNA breaks at transgenic loci inserted in the genome. Examples of reporter genes that allow the direct inspection of DSB repair pathways and transcription and chromatin dynamics are represented. Experimental approaches that can be coupled with the methods to induce DSBs at transgenic loci are shown.

The first reporter system, employing a site-specific DSB at a reporter transgene integrated into the genome of mammalian cells was developed in the mid-1990s. This genetic assay was devised by the Jasin lab to detect and quantify HR repair of DSBs induced by the rare-cutting endonuclease, I-*Sce*I (Rouet et al., 1994; Figure 3). Following this seminal work, a large number of labs further developed similar strategies based on I-*Sce*I cut of a transgenic locus to investigate various aspect of the DDR, including repair pathway preferences and efficiency (Gunn and Stark, 2012; Gelot et al., 2016), DNA-ends mobility and translocation (Soutoglou et al., 2007; Roukos et al., 2013), and the crosstalk with transcription (Shanbhag et al., 2010; Ui et al., 2015; Vítor et al., 2019). For example, Soutoglou et al. (2007) developed a cell system to visualize the dynamics of a single DSB induced at a defined genomic site in mammalian cells and demonstrated that broken ends are immobile in the nuclear space. For that, stable cell lines derived from mouse embryonic fibroblasts (NIH3T3) were generated containing a single I-*Sce*I restriction site flanked by arrays of lac-repressor binding sites and tetracycline-response elements (L-I-*Sce*I-T array) (Figure 3). Expression and binding of fluorescently-tagged lac and tetracycline-repressors to these arrays enabled the simultaneous detection of both DNA ends. The use of an I-*SceI* enzyme fused to a glucocorticoid receptor (I-*SceI*-GR) that translocated to the nucleus upon triamcinolone acetonide (TA) addition, allowed the controlled induction of a DSB at the L-I-*Sce*I-T array and the live-cell tracking of the broken DNA ends in real-time (Soutoglou et al., 2007).

Additional systems were further developed to generate multiple DSBs on a specific transgene, thus rendering the DNA repair easier to visualize. The Greenberg lab developed a noteworthy single-cell assay specifically designed to simultaneously analyze both the DSB repair and its effects on local transcription. The experimental procedure was based on the introduction of multiple nuclease-induced DSBs upstream the promoter of an inducible transgene, modified to enable the visualization of transcriptional and translational events (Shanbhag et al., 2010). The reporter system, integrated in the genome of a human osteosarcoma (U2OS) cell line, is visualized upon binding of the mCherry-fluorescently-tagged lac-repressor protein (mCherry-LacI) to a lac-operator array. Nascent transcription is visualized by the accumulation of fluorescent MS2-binding proteins at the transcription site, upon binding to nascent MS2 stem-loop structures present at the reporter gene RNA (Shanbhag et al., 2010). Expression of the *Fok*I nuclease domain fused to the mCherry-LacI creates DSBs at the lac operator array (Shanbhag and Greenberg, 2013). Of note, this approach leads to persistent and extensive DSB induction and the time of damage induction is dependent on the expression of mCherry-LacI-*Fok*I. A similar system to study transcription in proximity to DSBs was engineered by Ui et al. (2015). The authors established a U2OS cell line harboring multiple copies of an array of transcription units including tetracycline response elements (TRE) sites, MS2 sequences and I-*Sce*I restriction sites (Ui et al., 2015). Upon tamoxifen treatment, the mCherry-tTA-ER fusion proteins translocate into the nucleus and localize at transcription sites (TRE sites), to induce transcription activation, detected by the accumulation of fluorescently tagged-MS2 protein (Rafalska-Metcalf et al., 2010). Expression of a plasmid encoding I-*Sce*I generates DSBs at target restriction sites, thus enabling the study of the effect of DSBs on transcription. Using this experimental system the authors reported a DSB-induced transcriptional repression mechanism involving the transcription elongation factor ENL (Ui et al., 2015). More recently, the de Almeida lab developed a set of reporter genes that allow the direct visualization of transcription with single-molecule resolution upon the controlled induction of a unique DSB (Vítor et al., 2019; Figure 3). A single I-*Sce*I restriction site was inserted in either the promoter-proximal region or within an internal exon of a reporter gene. The binding of fluorescent proteins to MS2 and/or PP7 stem loops at the nascent transcripts allows measurements of transcription dynamics upon induction of the DSB. The exact timing of DSB induction is controlled using an I-*Sce*I-GR fusion protein. Using these reporters, the authors found that whereas induction of a DSB at the promoter region suppresses transcription, a DSB generated within an internal exon drives bidirectional break-induced transcription initiation (Vítor et al., 2019). In addition to live-cell microscopy imaging, these reporters may be combined with ChIP-qPCR, providing a valuable tool to directly inspect the recruitment of DNA repair factors to a DSB, to assess histone modifications or measure nucleosome occupancy at broken ends.

The direct visualization of DNA break-induced transcription activation using reporter genes, support a model whereby the DDR signaling involves the action of non-coding RNAs (ncRNAs) generated at sites of DNA damage (Michelini et al., 2018). To investigate the role of such DSB-induced ncRNAs in the DDR, the d’adda di Fagagna lab developed the RNase A treatment and reconstitution (RATaR) method, in which different RNA species of interest are used to reconstitute cells previously treated with recombinant RNase A (Michelini et al., 2019). RATaR may be employed to address the role of ncRNAs in the recruitment of repair proteins during the DDR using imaging approaches.

### Key Points

**(+)** I-*Sce*I or *Fok*I mediated DSB induction on transgenic loci are powerful systems to investigate the response to clean DSBs. These systems allow analyzing the repair event at a molecular resolution, the repair frequency (thanks to designed reporters cassettes) and the DNA damage repair/signaling in single cells (using imaging approaches). They can be combined with additional reporters to investigate with great detail the functional links between the DDR and transcriptional activity, chromatin modification and spatial organization, or DNA replication, for instance.

**(−)** These systems rely on transfection, transcriptional regulation, or nuclear localization of the endonuclease. Consequently, they cannot provide the same temporal resolution achieved using microirradiation, where DSB induction is immediate and highly synchronized. Moreover, the transgenic nature of the analyzed loci calls for caution, especially when repeat-rich transgenes are used (creating either multiple clustered DSBs or a single DSB but in a highly repeated transgenic locus, which may display a peculiar chromatin structure). Finally, the accurate repair of endonuclease-created breaks reconstitutes the target site, therefore being re-cleavable until the target site has been mutated. Hence, most of the outputs measured in these experimental contexts address mutagenic repair, leaving faithful repair out of reach.

## Methods to Induce DNA Breaks at Specific Endogenous Loci in the Genome

**FIGURE 4:**
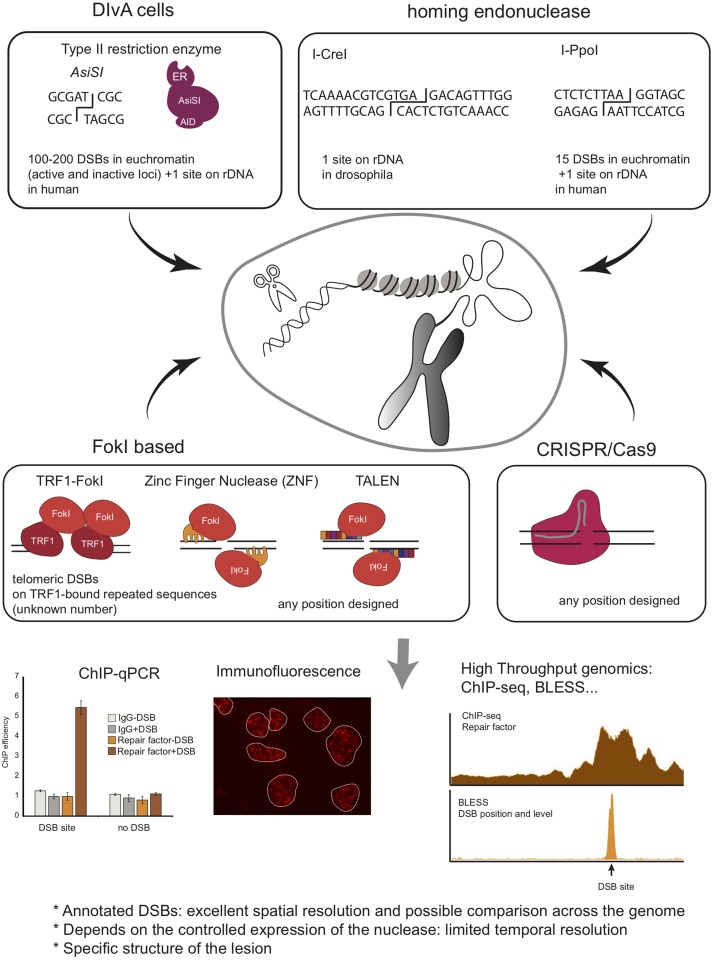
Schematic overview of methods to induce annotated DNA breaks at endogenous loci, including Type II endonuclease, Homing endonucleases, *Fok*I based system, and CRISPR/Cas9 system. Given that these DSBs are induced at annotated positions and in a homogeneous manner in the cell population, one can use ChIP to investigate protein recruitment at the site of damage. This can also be coupled to high throughput sequencing analyses to investigate simultaneously repair events at multiples breaks (ChIP-seq). Finally, BLESS, BLISS, Break-Seq and any other related genome wide methods to map DSB distribution across genomes can be used to analyze repair kinetics of these annotated DSBs.

In order to bypass the need for introducing a transgene and to avoid potential, non-generalizable, side effects of transgenic loci on the repair process (e.g., in the case of LacI repeats, a high copy number triggering a peculiar chromatin state), efforts have been made recently to develop alternative systems where DSBs can be induced at endogenous, annotated loci on the genome (Figure 4). On one hand, homing endonucleases and type II restriction enzymes have been used, allowing to induce breaks at annotated but not controllable positions, and on the other hand, the development of transcription activator-like effector nucleases (TALEN) and more recently of the clustered regularly interspaced short palindromic repeats (CRISPR)/Cas9 system has opened the possibility to introduce breaks at chosen loci.

### Type II Restriction Enzymes and Homing Endonucleases: Induction of Multiple DSBs but at Constrained Locations

#### I-*Ppo*I

The Kastan lab developed a system that uses the eukaryotic homing endonuclease I-*Ppo*I, which has a recognition sequence of 15bp, to form site-specific DSBs within endogenous target sites of the human genome (Berkovich et al., 2007). Expression of I-*Ppo*I in human cells results in the production of DSB at one site within the 28S ribosomal RNA gene, present in ∼300 copies, and fifteen additional unique loci. To tightly control DSB induction, a ligand-binding domain of the estrogen receptor (ER) was fused to I-*Ppo*I. The addition of 4-hydroxytamoxifen (4-OHT) promotes rapid nuclear localization of ER-I-*Ppo*I and the subsequent time-dependent cleavage of the endogenous sites. Using this system, the Kastan lab disclosed the distribution of Nbs1 and ATM, and histones (Berkovich et al., 2007; Goldstein et al., 2013) at DSBs by ChIP-qPCR. This system was used by others to investigate the dynamics of the transcription machinery following I-*Ppo*I DSB induction in RNA Polymerase II-transcribed genes, revealing a DNAPK-dependent break-induced transcriptional arrest (Pankotai et al., 2012; Caron et al., 2019), or to investigate the DDR induced in the nucleolus (Harding et al., 2015; Warmerdam et al., 2016; Pefani et al., 2018). I-*Ppo*I has been further applied to interrogate DSB repair mechanisms in other organisms, such as fission yeast (Sunder et al., 2012; Kuntz and O’Connell, 2013; Ohle et al., 2016) and mice (Kim et al., 2016). Of interest, in the latter, both temporal and spatial regulation of I-*Ppo*I activity was achieved by using a GFP-I-*Ppo*I endonuclease fused to an ER domain for tamoxifen-dependent temporal induction and whose tissue-specific expression was dependent on Cre recombinase. The results obtained using this *in vivo* model system showed transient, and DDR-dependent, decrease in gene expression of break-bearing - but not more distant - genes, further reversed upon DSB repair (Kim et al., 2016).

#### I-*Cre*I

In Drosophila, the I-*Cre*I homing endonuclease has also been used to create annotated DSBs in the ribosomal DNA (Royou et al., 2010). This allowed the authors to uncover a new Bub1R/Bub3/Polo kinase-dependent pathway that contributes to handle unrepaired rDNA DSBs during mitosis and to ensure correct segregation of broken chromosomes (Royou et al., 2010; Derive et al., 2015).

#### *Asi*SI

Another DSB-inducible tool developed to create multiple endogenous, sequence-specific breaks, makes use of the *Asi*SI - 8bp cutter - restriction enzyme. The Legube lab, fused *Asi*SI to a modified ER ligand-binding domain, which controls nuclear localization of *Asi*SI–ER fusion protein, and to an auxin-inducible degron enabling controlled ubiquitination and degradation of the enzyme (Iacovoni et al., 2010; Massip et al., 2010; Aymard et al., 2014). Stable integration of this construct in the genome of U2OS cells generated a DSB inducible via *Asi*SI (DIvA) system, where multiple annotated DSBs can be induced after 4-OHT treatment and DNA repair accurately monitored following auxin treatment. *Asi*SI induces 100-200 DSBs across the human genome [as determined by BLESS (Clouaire et al., 2018) and BLISS (Iannelli et al., 2017)], as well as one break in the ribosomal DNA repeat (Marnef et al., 2017). This system is then amenable to compare DNA repair at various genomic positions. Importantly, while *Asi*SI is not able to damage heterochromatin, likely due to both the DNA methylation status and decreased accessibility of compacted chromatin (Iacovoni et al., 2010; Clouaire et al., 2018), it induces DSBs at both transcribed and untranscribed loci (Aymard et al., 2014; Clouaire et al., 2018). The DIvA system has been used to inspect DNA repair pathway preferences at different chromatin regions (Aymard et al., 2014), to measure site-specific resection (Zhou et al., 2014) and repair kinetics and translocation frequency (Aymard et al., 2014; Cohen et al., 2018), and is instrumental to investigate the role of repair factors in HR or NHEJ [see, for example (Jacquet et al., 2016; Schrank et al., 2018)] Furthermore, combined with ChIP-seq, or any other high throughput genomic methods, it allows investigating DNA repair simultaneously at multiple DSBs and at high resolution. Consequently it has been extensively used to provide high-resolution maps of repair proteins and chromatin changes (Iacovoni et al., 2010; Caron et al., 2012, 2015; Aymard et al., 2014; Clouaire et al., 2018), of R-loops (Cohen et al., 2018; Lu et al., 2018), or long-range contacts (Aymard et al., 2017) around several breaks in the human genome. When combined to transcription mapping (RNA-seq, BrU-seq, Pol II ChIP-seq, or NET-seq) it disclosed insights into the interplay between γH2AX profile and transcription (Iacovoni et al., 2010), on the behavior of transcription at DSBs (Iacovoni et al., 2010; Iannelli et al., 2017; Cohen et al., 2018; Burger et al., 2019), as well as a novel DSB repair pathway coupled to transcription (Marnef et al., 2017).

#### Key Points

**(+)** These systems represent powerful tools to compare DNA repair events that occur at different genomic loci and, because they induce DSBs at annotated positions in a homogenous manner in the cell population, are compatible with all high resolution, high throughput sequencing-based techniques such as ChIP-seq, Hi-C, etc.

**(−)** For all these systems, as for the above-mentioned I-*Sce*I based systems: (i) DSB production is not immediate nor synchronized in the cell population and (ii) accurately repaired DSB can be re-cleaved. Hence while being powerful to analyze the spatial distribution of repair protein and chromatin changes around DSBs, they preclude a fine temporal resolution of these events. Moreover, the position of the DSBs is dictated by the target site of the chosen enzyme, which can represent a limitation to the number of different loci analyzed.

### Zinc Finger Nucleases, TALEN and CRISPR/Cas9: Induction of a Single DSB but at a Chosen Locus

A number of specific tools have more recently allowed to induce DSBs at chosen endogenous genomic loci.

#### Fusing *Fok*I to a Protein of Interest

To introduce DSBs at specific loci of interest, it is possible to fuse the *Fok*I endonuclease to a protein able to specifically target a particular locus. This approach was implemented for example to induce DSBs at telomeres by fusing *Fok*I to the shelterin protein TRF1 (Tang et al., 2013; Cho et al., 2014; Doksani and de Lange, 2016).

#### Zinc Finger Nucleases and TALEN

Zinc finger nucleases (ZNF) are chimeric proteins comprised of both a zinc finger domain designed to recognize a specific locus and the *Fok*I nuclease. Using a pair of ZNF binding opposite strands allows the introduction of a DSB at a locus of interest. For instance, ZNF able to target the intron 1 of the PPP1R12C gene (p84-ZNF) (Urnov et al., 2005) were used in order to investigate chromatin changes by ChIP-qPCR (Xu et al., 2012; Ayrapetov et al., 2014; Gursoy-Yuzugullu et al., 2015) and translocation biogenesis (Ghezraoui et al., 2014).

TALE proteins were discovered as composed of a succession of 34aa monomers, each displaying the ability to recognize one nucleotide. Fused to *Fok*I, this system provides a rapid and easy way to design sequence-specific nucleases called TALEN. TALEN have been used to investigate DNA repair in a large number of organisms and genomic contexts, such as in CTG trinucleotide repeats in budding yeast (Mosbach et al., 2018), or to understand the influence of the transcription status of a locus on the repair pathway choice (Aymard et al., 2014).

#### CRISPR/Cas9

The discovery of the CRISPR/Cas9 system in 2013 strongly revolutionized the DDR field by providing the ability to introduce DSBs at annotated loci, in a particularly simple and efficient manner, by the mean of a small guide RNA embedded in the Cas9 nuclease. For instance, this approach has been used successfully to induce DSBs and study DNA repair in rDNA (van Sluis and McStay, 2015; Korsholm et al., 2019). It allowed demonstrating RNA Pol I transcription inhibition *in cis* to rDNA DSBs and nucleolar reorganization upon rDNA breakage. CRISPR/Cas9 has also been instrumental to study the repair of heterochromatin. The Soutoglou lab used it to induce DSBs in a-satellites in mouse cells, demonstrating that, as for rDNA, heterochromatin foci are reorganized in G2 upon DSB induction (Tsouroula et al., 2016). CRISPR/Cas9 was also used to induce DNA breaks at multiple unique loci in order to study translocation biogenesis and repair mechanisms, such as on c-Myc, MLL, TMPRSS2, as well as G4 enriched or non-enriched genes (Ghezraoui et al., 2014; Day et al., 2017; Iannelli et al., 2017; Panchakshari et al., 2018; Wei et al., 2018; Meisenberg et al., 2019). However, of importance, it is yet unclear whether CRISPR/Cas9-induced breaks behave similarly to other types of DSBs. Indeed, recent studies indicated that Cas9-induced DSBs display highly mutagenic repair with nearly no accurate repair events (Brinkman et al., 2018; Richardson et al., 2018) and evidence suggests that they may be handled by the Fanconi Anemia repair pathway rather than canonical DSB repair machinery (Richardson et al., 2018).

#### Key Points

**(+)** Methods to induce DNA breaks at specific endogenous loci in the genome are particularly powerful in that they provide the liberty to choose the locus to be analyzed. As for the other endonuclease-mediated DSB induction systems, they are amenable to both imaging and molecular high throughput sequencing-based technologies such as ChIP-seq.

**(−)** Yet, similarly to the other endonuclease-mediated DSB induction, they are less suited for thorough, careful kinetics analyses since they rely on the controlled expression of the Cas9, or transient transfection of the sgRNA and accurately repaired DSBs may be re-cleaved by Cas9. Moreover, the fact that Cas9-induced DSBs may be particularly refractory to repair, and hence biased in terms of repair pathway choice, call for caution when using these systems.

### Telomere Deprotection as a Tool to Generate DSBs at Chromosome Ends

Coating of telomeres with shelterin factors including telomeric repeat-binding factor 2 (TRF2), prevents fusions of linear chromosome ends and suppresses local DNA damage responses (de Lange, 2018). Dysfunctional telomeres induce cellular responses that are highly similar to the ones elicited by DSBs, such as DDR activation and cellular senescence (Fumagalli et al., 2012; Hewitt et al., 2012). Indeed, replicative telomere shortening, which eventually culminates in telomere deprotection, induces molecular markers characteristic of DSBs and may serve as models to investigate DNA damage signaling in the context of senescence and aging (D’Adda Di Fagagna et al., 2003).

Dysfunctional telomeres can be generated through telomere uncapping and other forms of telomere damage, which may be specifically induced to activate the DDR in cycling cells. In addition to *Fok*I fusion with TRF1 described above, DSB-signaling at telomeres can be activated upon *TRF2* deletion (Celli and de Lange, 2005). Deletion of *TRF2* provokes sustained DNA damage at mammalian chromosome ends, and the resulting uncapped telomeres are processed by the NHEJ pathway (Celli and de Lange, 2005). A plethora of methods - ranging from the visualization of DNA repair factors foci using immunofluorescence to the biochemical characterization of DDR complexes assembled at dysfunctional telomeres using ChIP - can be coupled to the *TRF2* inactivation to investigate the molecular details of different aspects of the DDR. Importantly, dysfunctional telomeres have been instrumental to discover the function of various proteins in DSB repair [e.g., Rif1 (Zimmermann et al., 2013); Pol θ (Mateos-Gomez et al., 2015); the LINC complex (Lottersberger et al., 2015); or CST and shieldin (Mirman et al., 2018)].

#### Key Points

**(+)** Using dysfunctional telomeres as surrogates for DSBs is easy to implement and can be coupled with different imaging and biochemical approaches to directly inspect the molecular details of the DDR signaling.

**(−)** Telomeres possess several specific features that render them particularly refractory to repair, and, when uncapped through *TFR2* deletion, show a strong bias in terms of repair pathway choice toward NHEJ. The number of dysfunctional telomeres may vary considerably between cells and this heterogeneity may raise issues related with cells viability.

## Conclusion

Our capacity to create DSBs in a programed manner and in such a way that is compatible with a set of diverse methodologies to investigate the events that follow DNA damage, has led to our current deep understanding of the DDR. The induction of DSBs at random locations using different sources of radiation or genotoxic compounds, provides the easiest approach to analyze the recruitment kinetics of proteins to sites of DNA damage and is a powerful strategy to temporally resolve the sequence of DNA repair events. The development of methods to induce annotated DNA breaks at transgenic loci inserted in the genome, or at endogenous loci (restriction enzymes, CRISPR/Cas9) allowed the analysis of the DDR at molecular resolution and were instrumental in disclosing functional links between the DDR and processes such as transcriptional, chromatin dynamics, and DNA replication. Yet all the tools described here display significant drawbacks. For instance, nucleases-induced DSBs undergo consecutive cycles of repair/cleavage until these have been mutated, calling for caution when investigating DNA repair using these tools. A major challenge is now to refine these DSB-inducible systems and the subsequent methodologies to analyze repair in order to overcome these limitations.

## Author Contributions

All authors contributed to discussing the review contents and to writing the manuscript.

## Conflict of Interest

The authors declare that the research was conducted in the absence of any commercial or financial relationships that could be construed as a potential conflict of interest.
